# The Dundee Sanatorium Difficulty

**Published:** 1909-05-08

**Authors:** 


### The Dundee Sanatorium Difficulty.

The direct dependence of anti-tuberculosis measures upon economic conditions is well shown by the difference of opinion in connection with the proposed municipal sanatorium for Dundee, which has aroused considerable public feeling in the district. Certainly this community is not a wealthy one; but then the chance has recently been, and is still, open to it of securing an existing sanatorium upon advantageous terms. Moreover, it cannot be said that the need is not urgent, since there are nearly two thousand consumptives in the city, and the general physical condition of its people, as judged by the index of the percentage of rejections in Army recruiting, has repeatedly appeared to be in a deplorable condition. The opponents of the scheme of taking over and municipalising the Sidlaw sanatorium have themselves used the words " valuable gift " in connection with the transaction, and further estimate the value of the property at £10,000. After this, it seems unreasonable to stand out for the endowments of that institution—some £19,000—being handed over to the Town Council in addition to the property. The sanatorium, it appears, contains at the present time forty available beds, and the plan favoured by the Lord Provost and Town Council of Dundee is to raise this number to 150, for forty of which a charge of 10s. a week each is to be made. It is fairly evident, however, both from a circular issued by the Dundee Citizens' Union and from reports in the *Dundee Advertiser* of April 20 of a very lively public meeting convened to consider the question, that in the present state of local opinion the plan stands little chance of being adopted; indeed, the Local Government Board has stated that, in view of numerous important representations against the scheme, it cannot, without further consideration, give its statutory sanction as yet. But if the popular verdict is clear, the same cannot be said of the reasons advanced by the popular spokesmen. To anyone conversant with the anti-tuberculosis campaign their case will appear much overstated. They quote official figures to the effect that there are from 1,500 to 2,000 cases of phthisis in Dundee, and ask whether 150 beds will be enough. Now it is, unfortunately, probable that these will quite suffice for the proportion of cases which present a good prospect of economic cure. The popular leaders doubt whether paying patients will consent to be treated in a charitable institution, forgetting that illness and fear of death have ever a stilling influence on social prejudices, and that sanatorium treatment at 10s. a week is a very good bargain for the phthisical patient —and so on and so on. The real crux of the matter—Is Dundee financially equal to the maintenance of a municipal sanatorium?—can, of course, only be judged of by its inhabitants; but it is not indicative of an unprejudiced and fair outlook that Dr. Sinclair's suggestion of a middle course (municipalising the Sidlaw Sanatorium and working it on its present scale) has received only passing mention and attracted no discussion at all. It is a pity that this, the first endeavour to follow the good example set by Birmingham to British municipal authorities, should have aroused such opposition, especially since the preliminary financial obstacles seem here to have been smoothed away to some extent. But the present deadlock is what must be expected to occur from time to time. The work of science—in this particular case of applied science in the shape of medicine—has in the last few decades revealed new knowledge, new possibilities, unsuspected responsibilities; and society in general, while accepting the pleasant results at the easy price of a little perfunctory praise, shows little inclination to bear its share of the burdens. What experienced and politically unprejudiced sanitarians are openly saying is that economic conditions rather than medical demands will have to be modified if progress is to be maintained. It is not at all certain that this estimate of their own importance is exaggerated; and we still hope that the growing education of the public in medical and hygienic matters will gradually produce that ideal co-operation between the medical profession and the general public to which we have so long and so patiently looked forward.

